# Supplementary material for: Human mitochondria require mtRF1 for translation termination at non-canonical stop codons
Source: Nat Commun. 2023 Jan 3;14:30. doi: 10.1038/s41467-022-35684-6 (PMC9810596; doi:10.1038/s41467-022-35684-6)
Supplement: Supplementary file 1 — Supplementary Information [file 41467_2022_35684_MOESM1_ESM.pdf]

SUPPLEMENTARY INFORMATION

Human mitochondria require mtRF1 for translation termination at non-canonical stop codons

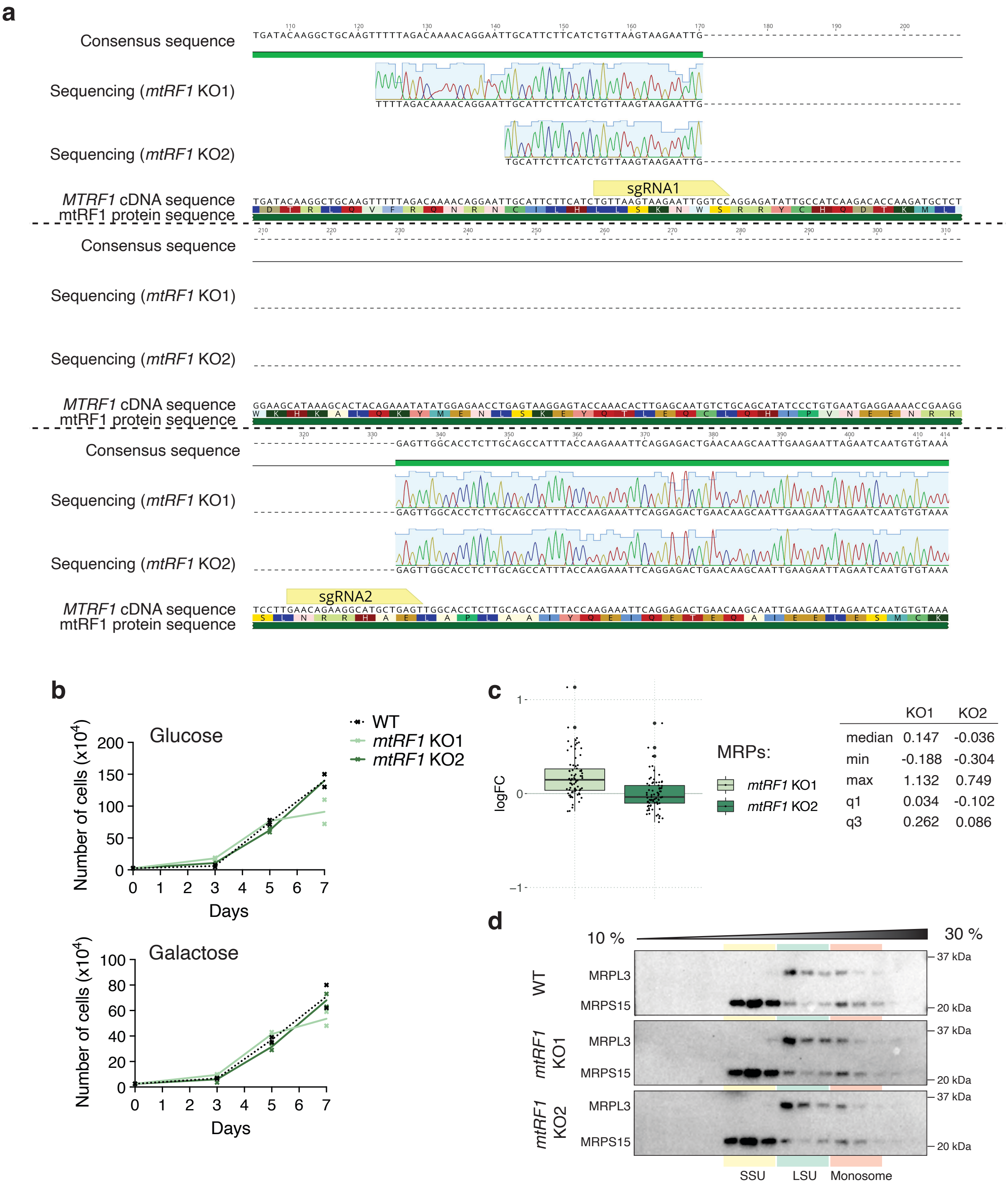

Supplementary Fig. 1: Analysis of *mtRF1* KO cells. **a** Out-of-frame deletion within *MTRF1* generated by CRISPR/Cas9 system analyzed by Sanger sequencing. Alignments of sequencing data and *MTRF1* cDNA sequence are depicted. **b** Growth curve of HEK WT and *mtRF1* KO cells in glucose and galactose based medium. 5 x 10^4 cells were seeded on day 0. Two biological replicates are plotted for each sample. **c** Quantitative mass spectrometry analysis of mitoribosomal proteins (MRPs) from WT and *mtRF1* KO mitolysates. Log2(fold change) (logFC) of *mtRF1* KO1 and *mtRF1* KO2 compared to WT samples are plotted (values are listed in Supplementary Table 3). Each dot represents one MRP. n=3 independent experiments. **d** Sedimentation of mitochondrial ribosomes from WT and *mtRF1* KO cells on 10–30 % isokinetic sucrose gradients. Equal amounts of mitolysates were loaded. 100 µl fractions were collected

and analyzed by western blotting using antibodies against mitochondrial LSU (MRPL3) and SSU (MRPS15). Expected fractions containing SSU, LSU and monosome are highlighted. A representative blot of n=2 technical replicates is depicted.

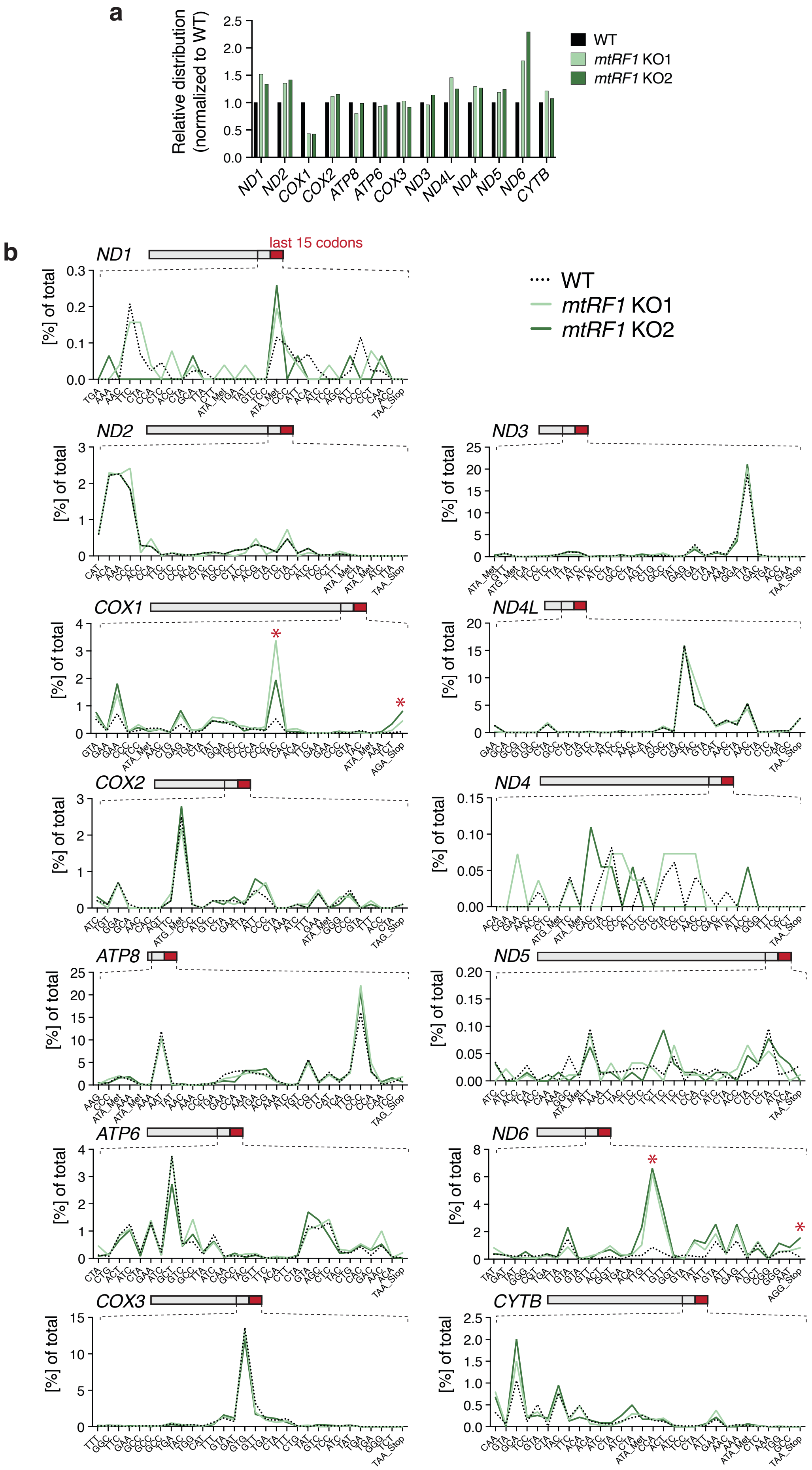
a
Relative distribution (normalized to WT)
WT
mtRF1 KO1
mtRF1 KO2
ND1
ND2
COX1
COX2
ATP8
ATP6
COX3
ND3
ND4L
ND4
ND5
ND6
CYTB
b
last 15 codons
[%] of total
WT
mtRF1 KO1
mtRF1 KO2
*

Supplementary Fig. 2: Analysis of ribosome profiling data of WT and *mtRF1* KO cells. **a** Percentual occupancies of mitoribosomes from *mtRF1* KO cells on mitochondrial transcripts normalized to WT (refers to Fig. 1b). This graph does not show absolute occupancy differences but relative distributions. **b** Occupancy of mitoribosomes within the last 30 codons of each ORF relative to the total occupancy on each transcript. The length of each transcript is shown schematically, where the length of the depicted area is highlighted in red. Stalling was observed for COX1 and ND6 (marked with *).

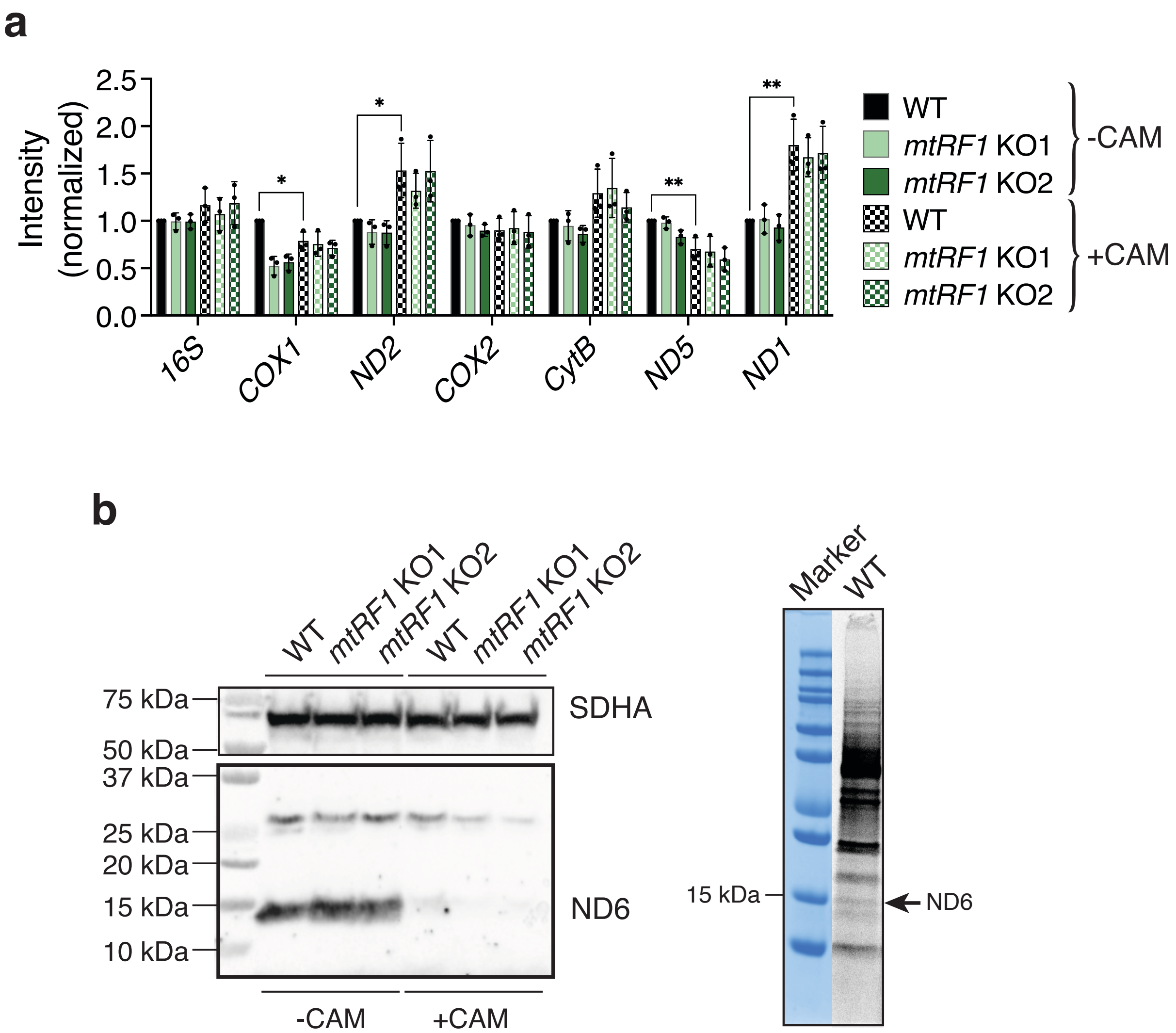

Supplementary Fig. 3: a Quantification of Fig. 2c samples normalized to WT (-CAM) samples. To account for different loading, all band intensities were first normalized to 18S rRNA band intensity (EtBr staining). Means and SD of n=3 independent experiments. Unpaired two-tailed t-test (* P<0.05; ** P<0.01; *** P<0.001; significances for *mtRF1* WT -/+ CAM are shown). **b** Evaluation of ND6 migration in SDS-PAGE. Left: ND6 antibody was tested on mitolysates from cells non-treated/treated for 48 h with chloramphenicol. Loading was assessed by SDHA detection. Same blot as in Fig. 2g (right) is shown. Right: *De novo* synthesis of mitochondrial proteins from WT cells analyzed by [^{35}S]-labeling. Radiograph was aligned with protein marker from the same gel. Band corresponding to ND6 was assigned according to western blot results. n=1 experiment.

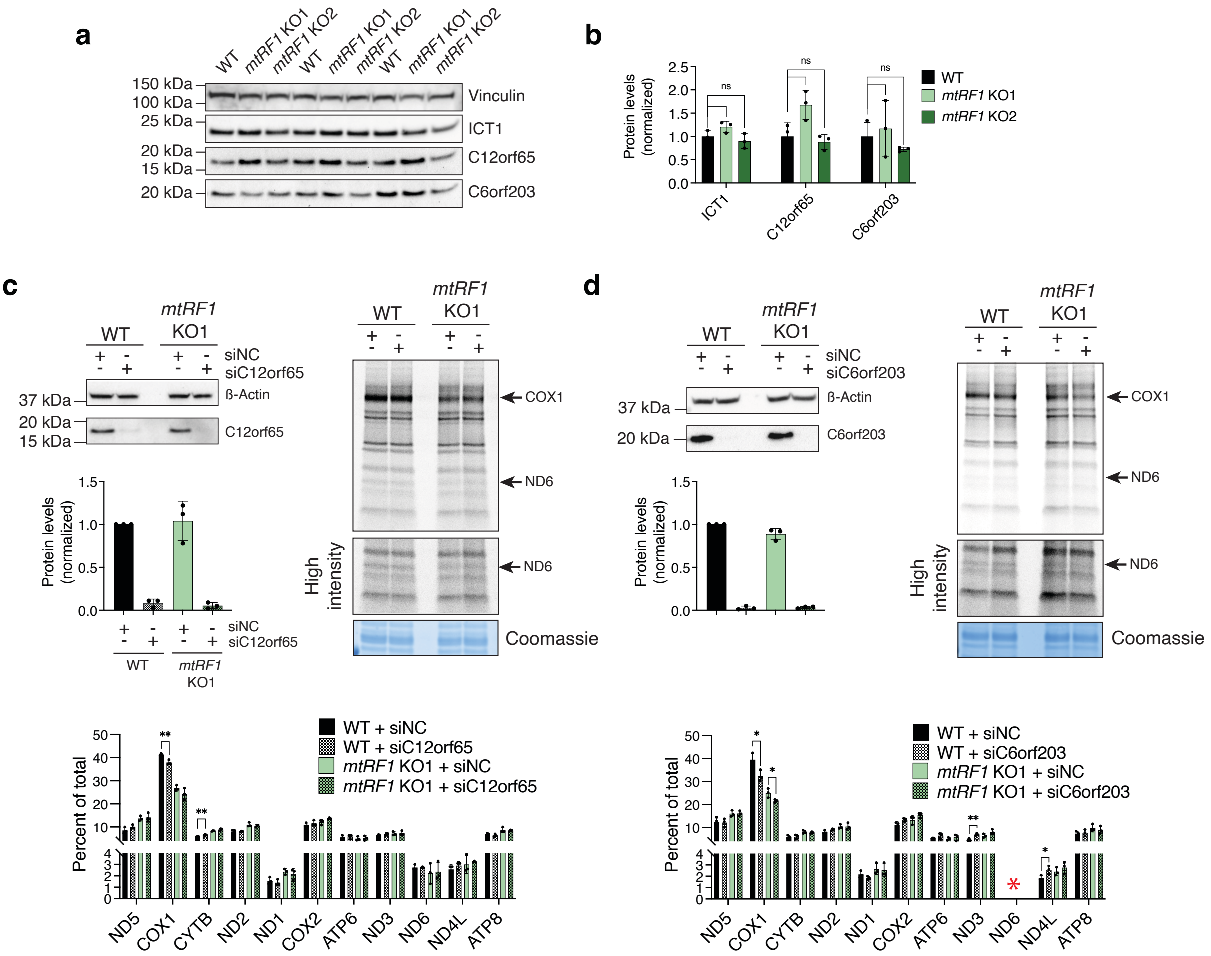

Supplementary Fig. 4: Analysis of components of the mitoribosome rescue machinery upon loss of mtRF1. **a** ICT1, C12orf65, and C6orf203 protein levels analyzed by western blotting. Whole cell lysates were loaded. Loading was assessed by Vinculin detection. **b** Quantification of a) normalized to protein levels in WT cells. Means and SD of n=3 replicates. Unpaired t-test (* P<0.05; ** P<0.01; *** P<0.001). **c** Knockdown of C12orf65 in WT and *mtRF1* KO1 cells. Cells were either transfected with siRNA targeting *C12orf65* (si*C12orf65*) or non-targeting control (siNC). 72 h after transfection C12orf65 protein steady-state levels were analyzed by western blotting (Upper left: Representative blot and quantification of C12orf65 protein levels normalized to WT + siNC from three independent experiments). *De novo* synthesis of mitochondrial proteins was analyzed by [^{35}S]-labeling (Upper right: Representative image of three independent experiments; arrows highlight bands corresponding to COX1 and ND6). Lower center: Quantification of [^{35}S]-labeling normalized to the total signal of the respective sample. Means and SD of n=3 independent experiments. Paired two-tailed t-test (* P<0.05; ** P<0.01; *** P<0.001).). **d** Knockdown of *C6orf203* in WT and *mtRF1* KO1 cells. Cells were either transfected with siRNA targeting *C6orf203* (si*C6orf203*) or non-targeting control (siNC). 72 h after transfection C6orf203 protein steady state levels were analyzed by western blotting (Upper left: Representative blot and quantification of C6orf203 protein levels normalized to WT + siNC from three independent experiments). *De novo* synthesis of mitochondrial proteins was analyzed by [^{35}S]-labeling (Upper right: Representative image of three independent experiments; arrows highlight bands corresponding to COX1 and ND6). Lower center: Quantification of [^{35}S]-labeling normalized to the total signal of the respective sample. Means and SD of n=3 independent experiments. Due to low resolution, ND6 could not be quantified (highlighted by red asterisk). Paired two-tailed t-test (* P<0.05; ** P<0.01; *** P<0.001).

a

	Complex I		Complex II		Complex III		Complex IV		Complex V	
	KO1	KO2	KO1	KO2	KO1	KO2	KO1	KO2	KO1	KO2
median	-0.007	-0.025	-0.306	0.198	0.118	-0.143	-0.712	-0.857	0.126	0.045
min	-0.369	-0.481	-0.76	0.1	-0.128	-0.274	-0.976	-1.463	-0.243	-0.063
max	0.783	0.5	0.148	0.296	0.635	0.549	-0.167	-0.134	1.194	0.901
q1	-0.178	-0.132	-0.533	0.149	0.028	-0.188	-0.918	-1.08	-0.141	-0.007
q3	0.169	0.101	-0.079	0.247	0.276	0.043	-0.68	-0.561	0.219	0.072

b

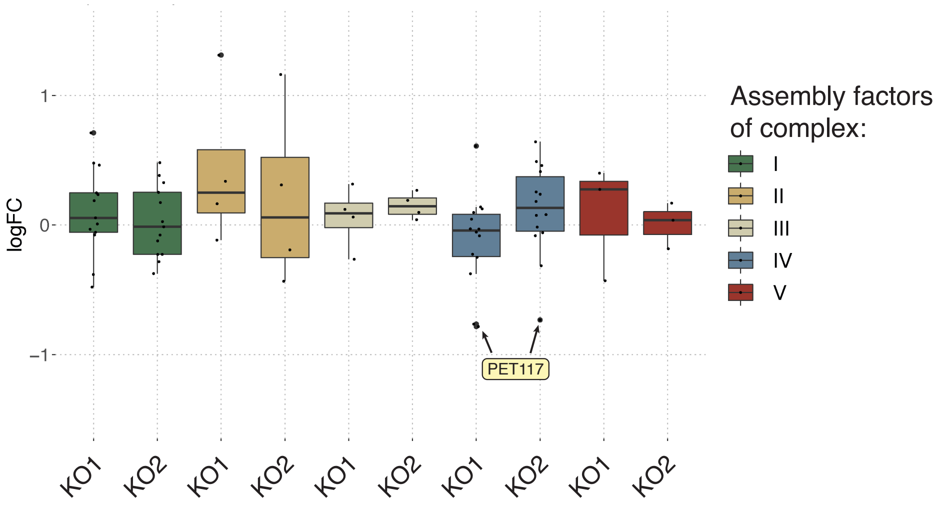

	Complex I		Complex II		Complex III		Complex IV		Complex V	
	KO1	KO2	KO1	KO2	KO1	KO2	KO1	KO2	KO1	KO2
median	0.055	-0.014	0.25	0.058	0.09	0.144	-0.044	0.131	0.274	0.037
min	-0.479	-0.375	-0.117	-0.434	-0.264	0.04	-0.784	-0.733	-0.43	-0.184
max	0.711	0.481	1.311	1.161	0.315	0.267	0.609	0.642	0.4	0.168
q1	-0.057	-0.227	0.094	-0.252	-0.02	0.083	-0.244	-0.049	-0.078	-0.074
q3	0.248	0.252	0.581	0.522	0.169	0.209	0.083	0.373	0.337	0.103

Supplementary Fig. 5: a Summary of boxplots presented in Fig. 3a. **b** Quantitative mass spectrometry analysis of OXPHOS assembly factors from mitolysates from *mtRF1* KO1 and KO2 compared to WT cells. Log2(fold change) (logFC) compared to WT samples is plotted (values are listed in Supplementary Table 5). Each dot represents one assembly factor. Same dataset as in Fig. 3a. n=3 independent experiments.

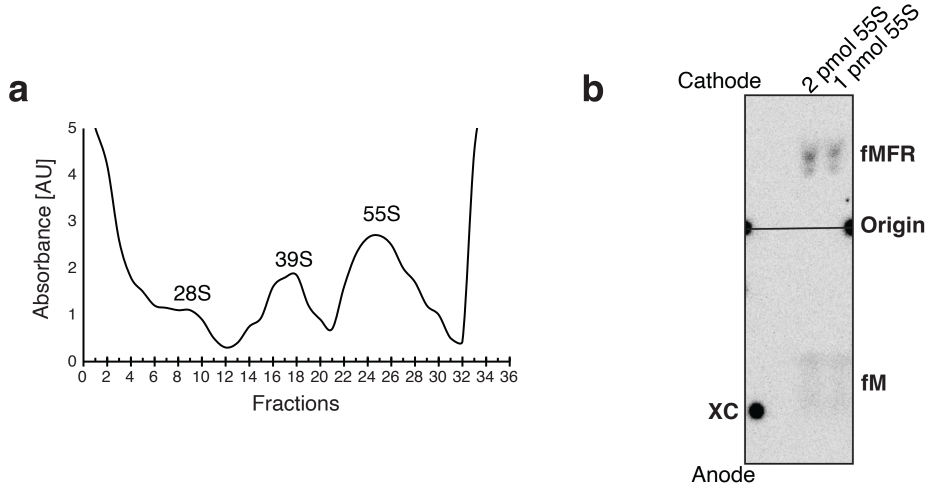

Supplementary Fig. 6: *In vitro* mitochondrial translation assay. **a** Purification of liver mitochondrial ribosomes. Fractions 24-28 containing the full monosomes were pooled and used for *in vitro* translation experiments. **b** Analysis of the fM*FR tripeptide by thin layer electrophoresis. The radioactively labelled tripeptide was located by autoradiography and quantified by scintillation counting.

Supplementary Table 1: Oligonucleotides used in the study

Oligonucleotide name	Sequence (5`->3`)	Note
SgRNA1 top	CACCGTGTTAAGTAAGAATTGGTCC	Generation *mtRF1* KO cells
SgRNA1 bottom	AAACGGACCAATTCTTACTTAACAC	Generation *mtRF1* KO cells
SgRNA2 top	CACCGGAACAGAAGGCATGCTGAGT	Generation *mtRF1* KO cells
SgRNA2 bottom	AAACACTCAGCATGCCTTCTGTTCC	Generation *mtRF1* KO cells
KO PCR primer FWD	ACCTCCAGTGTCACATCCAG	Analysis *mtRF1* KO cells
KO PCR primer REV	CTGTACTTACACACACGCATATAAGTG	Analysis *mtRF1* KO cells
BamHI-mtRF1-FLAG-F	CTTTCTTGGATCCATGAATCGTCACCTGTGTGTTTGG	Used to clone mtRF1 ORF with C-terminal FLAG tag into pcDNA5/FRT/TO
mtRF1-FLAG-XhoI-R	AAGAAAGCTCGAGCTACTTATCGTCGTCATCCTTGTAATCTTTTGCTGATTTAAGGTGTTCATCC	Used to clone mtRF1 ORF with C-terminal FLAG tag into pcDNA5/FRT/TO
mtRF1-noFLAG-F	TAGCTCGAGTCTAGAGGG	Used to remove C-terminal FLAG tag from the cloned construct
mtRF1-noFLAG-R	TTTTGCTGATTTAAGGTGTTCATCC	Used to remove C-terminal FLAG tag from the cloned construct
mtRF1-AAQ-mut-F	**GCCGCCCAG**CATGTTAATAAAACTGATAGTGCCG	Used to create AAQ mutant mtRF1 variant; bold part represents codons for AAQ motif
mtRF1-AAQ-mut-R	TGCTCCTTTGGCTCG	Used to create AAQ mutant mtRF1 variant
CMV forward primer	CGCAAATGGGCGGTAGGCGTG	Used to sequence inserts in pcDNA5/FRT/TO-based constructs
BGH reverse primer	TAGAAGGCACAGTCGAGG	Used to sequence inserts in pcDNA5/FRT/TO-based constructs
3' adapter (RA3)	TGGAATTCTCGGGTGCCAAGG	Illumina, cat#RS-200-0012
5' adapter (RA5)	GUUCAGAGUUCUACAGUCCGACGAUC	Illumina, cat#RS-200-0012
RT primer (RTP)	GCCTTGGCACCCGAGAATTCCA	Illumina, cat#RS-200-0012
RNA PCR primer 1 (RP1)	AATGATACGGCGACCACCGAGATCTACACGTTCAGAGTTCTACAGTCCGA	Illumina, cat#RS-200-0012
RNA PCR primer 2 (RP2)	CAAGCAGAAGACGGCATACGAGATCGTGATGTGACTGGAGTTCCTTGGCACCCGAGAATTCCA	Illumina, cat#RS-200-0012
NB_ND3_F	ATAAACTTCGCCTTAATTTTAATAATC	Used to produce gene-specific DNA template for northern blotting probe, same for oligonucleotides below

NB_ND3_R	**TAATACGACTCACTATAGGGA**TTCGGTTCAGTCTAATCCTTTTTGTAG	Bold part represents T7 promoter
NB_CYTB_F	CTACCTTCACGCCAATGG	
NB_CYTB_R	TTTGTTAGGGACGGATCG	
NB_ND1_F	AACCTCAACCTAGGCCTCC	
NB_ND1_R	AATGCTAGGGTGAGTGGTAGG	
NB_COX1_F	CTTATTCGAGCCGAGCTG	
NB_COX1_R	GGTATAGAATGGGGTCTCCTC	
NB_COX3_F	CCACCAATCACATGCCTATC	
NB_COX3_R	ACGTGAAGTCCGTGGAAGCC	
NB_ND4_F	ACTACCACTGACATGACTTTCC	
NB_ND4_R	GGAGTCATAAGTGGAGTCCG	
NB_COX2_F	GCGCAAGTAGGTCTACAAGACGC	
NB_COX2_R	GCATGAAACTGTGGTTTGCTCC	
NB_ND2_F	TCCCAGAGGTTACCCAAG	
NB_ND2_R	GAGTAGTGTGATTGAGGTGGAG	
NB_ATP8/6_F	CCCATACTCCTTACACTATTCC	
NB_ATP8/6_R	GTTAGCGGTTAGGCGTAC	
NB_ND5_F	GTAGCATTGTTCGTTACATGG	
NB_ND5_R	ACTGCTGCGAACAGAGTG	
NB_ND6_F	GGGGTTTTCTTCTAAGCCTTC	
NB_ND6_R	**TAATACGACTCACTATAGGG**CCCCCGAGCAATCTCAATTAC	Bold part represents T7 promoter
NB_12S_F	CACTGAAAATGTTTAGACGGG	
NB_12S_R	GGCTCCTCTAGAGGGATATG	
NB_16S_F	TAGATATAGTACCGCAAGGG	
NB_16S_R	GACTTGTTGGTTGATTGTAG	
mRNA sequence mitochondrial translation elongation	AUG UUC AGA CAA UUC AGA CAA GCG GUA GGU CUA CAA CAA GUA CAU UCA AAA UCC AAC	In vitro translation assay
mRNA sequence mitochondrial translation termination UAG	AUG CAA UAG UGG UUC AGA CAA GCG GUA GGU CUA CAA CAA GUA CAU UCA AAA UCC AAC	In vitro translation assay
mRNA sequence mitochondrial translation termination AGA	AUG CAA AGA UGG UUC AGA CAA GCG GUA GGU CUA CAA CAA GUA CAU UCA AAA UCC AAC	In vitro translation assay
mRNA sequence mitochondrial translation termination AGG	AUG CAA AGG UGG UUC AGA CAA GCG GUA GGU CUA CAA CAA GUA CAU UCA AAA UCC AAC	In vitro translation assay
mRNA sequence cytoplasmic translation elongation	GGATCCTAATACGACTCACTATAGGGAGACCGGAATTCAAAGCAAAAATGTGATCTTGCTTGTAAATACAATTTTGAGAGGTTAATAAATTACAAGTAGTGCTATTTTTGTATTTAGGTTAGCTATTTAGCTTTACGTTCCAGGATGCCTAGTGGCAGCCCCACAATATCCAGGAAGCCCTCTCTGCGGTTTTTCAGATTAGGTAGTCGAAAAACCTAAGAAATTTACCT TTCAAAGTGAGACAATGGCTAATGACATTTCA	In vitro translation assay

	AGATACCATGGAAGACGCCAAAAACATA AAGAAAGGCCCGGAAGCTT	

Supplementary Table 2: Antibodies used in the study

Antibodies	Dilution	Source	Identifier
MRPL3	1:1000	Sigma-Aldrich	RRID: AB_2678606; Cat#HPA043665
MRPS15	1:2000	Proteintech Group	RRID: AB_2301068; Cat#17006-1-AP
OXPHOS cocktail human	1:1000	Abcam	Cat#ab110411
mtRF1	1:1000	Sigma-Aldrich	RRID: AB_10795038; Cat# HPA043316
SDHA	1:2000	Invitrogen	RRID: AB_1501830; Cat#459200
COX1	1:1000	Invitrogen	RRID: AB_ 2532240 ; Cat#459600
ND6	1:500	Thermo Fisher	RRID: AB_2855404; Cat#PA5-109993
Vinculin	1:1000	Cell Signaling	RRID: AB_10559207; Ca#4650
Beta-Actin	1:1000	Abcam	RRID: AB_306371;Cat#ab8226
NDUFB10	1:500	Abcam	Cat#ab196019
NDUFA9	1:1000	Abcam	RRID: AB_301431; Cat#ab14713
COX2	1:1000	Abcam	RRID: AB_10887758; Cat#ab110258
COX5a	1:1000	Molecular Probes	RRID: AB_1501848; Cat# A21363
Anti-rabbit IgG F(ab')$_2$-HRP	1:5000	GE Healthcare	RRID: AB_772191; Cat#NA9340
Sheep Anti-Mouse IgG, Whole Ab ECL Antibody, HRP conjugated	1:5000	GE Healthcare	RRID: AB_772209; Cat#NXA931

Supplementary Table 3: MS data MRPs

Gene name	logFC KO2 vs. WT	logFC KO1 vs. WT
MRPL2	0.2	1.132
MRPL44	0.061	0.704
MRPS2	-0.262	-0.186
MRPL37	-0.18	-0.149
MRPL32	0.749	0.204
MRPS24	-0.015	0.139
MRPL4	-0.044	0.061
MRPS5	0.008	0.015
MRPL15	0.12	0.461
MRPL38	-0.176	0.044
DAP3	0.12	0.026
MRPL22	0.13	0.537
MRPS21	0.056	0.166
MRPS6	0.031	0.119
MRPS23	-0.073	0.003
MRPL51	0.011	0.52
MRPL46	0.118	0.291
MRPL1	-0.215	0.001
MRPL48	-0.103	-0.04
MRPS33	-0.042	0.103

MRPL13	-0.236	-0.018
MRPS14	-0.009	0.153
MRPS30	-0.136	-0.008
MRPL16	-0.158	0.248
MRPS18C	-0.1	-0.038
MRPS26	-0.092	0.189
MRPL20	-0.059	0.034
MRPS34	-0.124	-0.089
MRPL35	-0.063	0.379
MRPL28	-0.165	0.04
MRPS17	-0.036	0.018
MRPL57	-0.067	0.158
MRPS16	0.489	0.591
MRPL34	0.17	0.483
AURKAIP1	-0.11	0.11
MRPL3	0.087	0.197
MRPL40	0.105	0.502
MRPS31	-0.073	-0.046
MRPL10	-0.128	-0.02
MRPL50	-0.082	0.048
MRPS12	0.287	0.569
MRPL42	-0.069	-0.013
MRPS27	0.058	0.04
MRPL33	-0.09	0.035
MRPS15	0.398	0.558
MRPL24	-0.099	0.165
MRPL21	-0.304	-0.12
MRPL23	-0.264	0.046
MRPL18	-0.22	0.055
MRPS18A	-0.085	0.156
MRPS25	-0.006	0.074
MRPL39	-0.066	0.242
CHCHD1	0.246	-0.188
MRPL30	0.023	0.14
MRPL11	-0.037	0.107
MRPL27	-0.131	0.094
MRPL54	-0.24	0.057
MRPL41	-0.17	0.239
MRPL55	-0.068	0.156
MRPS18B	0.111	0.306
MRPL43	-0.224	0.239
MRPS10	0.151	0.241

MRPS11	0.122	0.267
MRPL9	0.157	0.545
MRPS35	0.024	0.134
MRPL49	0.083	0.302
MRPS22	0.292	0.477
MRPS28	0.051	0.216
MRPL14	0.005	0.41
MRPL53	-0.049	-0.034
MRPL45	-0.018	0.187
MRPL17	0.088	0.249
MRPL19	-0.014	0.186

Supplementary Table 4: MS data OXPHOS complexes

Gene name	Complex	logFC KO2 vs. WT	logFC KO1 vs. WT
NDUFA7	I	-0.025	-0.002
NDUFA12	I	0.055	-0.016
NDUFC2	I	-0.165	-0.22
NDUFS5	I	0.122	0.029
NDUFB7	I	0.081	0.023
NDUFB5	I	-0.481	-0.219
NDUFA13	I	-0.058	-0.265
NDUFB1	I	0.094	-0.006
NDUFA2	I	-0.228	-0.178
NDUFS4	I	0.003	0.21
NDUFS3	I	-0.073	-0.141
NDUFA10	I	-0.134	-0.117
NDUFB4	I	-0.132	-0.13
NDUFB9	I	-0.038	0.296
NDUFS6	I	0.051	-0.189
NDUFB2	I	-0.407	0.239
NDUFA9	I	-0.224	-0.369
NDUFA5	I	-0.06	-0.007
NDUFB10	I	0.24	0.169
NDUFA8	I	0.125	0.003
NDUFB3	I	0.101	-0.163
NDUFC1	I	0.349	0.182
NDUFB11	I	-0.088	-0.276
NDUFB6	I	0.403	0.169
NDUFV3	I	0.5	0.783
SDHB	II	0.1	0.148
SDHA	II	0.296	-0.76
UQCRFS1	III	-0.159	0.08

UQCRQ	III	-0.274	-0.128
UQCRH	III	0.549	0.635
UQCRB	III	-0.126	0.156
COX5B	IV	-0.993	-0.919
COX6C	IV	-1.039	-0.976
COX6B1	IV	-0.579	-0.72
NDUFA4	IV	-1.463	-0.703
COX7A2	IV	-1.202	-0.917
COX7C	IV	-0.721	-0.636
COX5A	IV	-0.507	-0.695
COX4I1	IV	-0.134	-0.167
MT-ATP8	V	0.025	0.219
MP68	V	-0.063	-0.243
ATPIF1	V	0.241	1.194
ATP5J	V	0.045	0.161
ATP5J2	V	-0.053	-0.195
ATP5O	V	-0.007	0.262
ATP5E	V	0.901	0.987
ATP5D	V	0.072	0.023
ATP5F1	V	-0.018	-0.141
ATP5A1	V	0.114	0.126
ATP5B	V	0.046	-0.168
ATP5H	V	0.018	0.164
ATP5C1	V	0.069	-0.076

Supplementary Table 5: MS data OXPHOS assembly factors

Gene name	Complex	logFC KO2 vs. WT	logFC KO1 vs. WT
ATP5SL	I	0.327	0.248
TIMMDC1	I	0.173	0.478
C17orf89	I	0.252	0.711
NDUFAF7	I	-0.227	0.234
NDUFAF5	I	-0.375	-0.479
NDUFAF3	I	-0.014	-0.382
NDUFAF4	I	-0.123	0.055
NDUFAF2	I	0.481	0.462
TMEM126B	I	0.382	-0.057
ECSIT	I	0.026	0.187
NDUFAF1	I	-0.227	-0.077
ACAD9	I	-0.078	-0.033
LYRM2	I	-0.283	0.008
SDHAF2	II	0.309	0.164
SDHAF4	II	1.161	1.311

SDHAF1	II	-0.192	-0.117
SDHAF3	II	-0.434	0.337
LYRM7	III	0.04	0.12
UQCC2	III	0.267	0.315
TTC19	III	0.19	0.061
BCS1L	III	0.097	-0.264
COA5	IV	-0.315	-0.784
PET117	IV	-0.733	-0.765
CMC1	IV	-0.06	-0.377
PNKD	IV	0.255	0.096
COX18	IV	0.459	-0.084
COX20	IV	-0.016	-0.25
CMC2	IV	0.642	0.124
CEP89	IV	0.412	0.609
SURF1	IV	-0.083	-0.055
CMC2	IV	0.182	-0.03
COA4	IV	0.489	-0.032
SMIM20	IV	0.074	0.045
COA7	IV	0.08	-0.226
COA6	IV	0.237	0.139
ATPAF1	V	0.037	0.274
FAM173B	V	0.168	-0.43
C7orf55	V	-0.184	0.4

Supplementary Table 6: TMT labeling scheme

tmt_label	Genotype	Replicate	Sample
126	WT	1	WT_1
127N	WT	2	WT_2
127C	WT	3	WT_3
128N	MtRF1_KO2	1	MtRF1_KO2_1
128C	MtRF1_KO2	2	MtRF1_KO2_2
129N	MtRF1_KO2	3	MtRF1_KO2_3
131N	MtRF1_KO1	1	MtRF1_KO1_1
131C	MtRF1_KO1	2	MtRF1_KO1_2
132N	MtRF1_KO1	3	MtRF1_KO1_3
