# Supplementary material for: Human mitochondria require mtRF1 for translation termination at non-canonical stop codons
Source: Nat Commun. 2023 Jan 3;14:30. doi: 10.1038/s41467-022-35684-6 (PMC9810596; doi:10.1038/s41467-022-35684-6)
Supplement: Supplementary file 3 — Reporting Summary [file 41467_2022_35684_MOESM3_ESM.pdf]

Corresponding author(s): Joanna Rorbach

Last updated by author(s): Dec 5, 2022

# Reporting Summary

Nature Portfolio wishes to improve the reproducibility of the work that we publish. This form provides structure for consistency and transparency in reporting. For further information on Nature Portfolio policies, see our Editorial Policies and the Editorial Policy Checklist.

## Statistics

For all statistical analyses, confirm that the following items are present in the figure legend, table legend, main text, or Methods section.

| n/a | Confirmed | |
|---|---|---|
| ☐ | ☒ | The exact sample size (*n*) for each experimental group/condition, given as a discrete number and unit of measurement |
| ☐ | ☒ | A statement on whether measurements were taken from distinct samples or whether the same sample was measured repeatedly |
| ☐ | ☒ | The statistical test(s) used AND whether they are one- or two-sided<br>*Only common tests should be described solely by name; describe more complex techniques in the Methods section.* |
| ☒ | ☐ | A description of all covariates tested |
| ☒ | ☐ | A description of any assumptions or corrections, such as tests of normality and adjustment for multiple comparisons |
| ☐ | ☒ | A full description of the statistical parameters including central tendency (e.g. means) or other basic estimates (e.g. regression coefficient) AND variation (e.g. standard deviation) or associated estimates of uncertainty (e.g. confidence intervals) |
| ☐ | ☒ | For null hypothesis testing, the test statistic (e.g. *F*, *t*, *r*) with confidence intervals, effect sizes, degrees of freedom and *P* value noted<br>*Give P values as exact values whenever suitable.* |
| ☒ | ☐ | For Bayesian analysis, information on the choice of priors and Markov chain Monte Carlo settings |
| ☒ | ☐ | For hierarchical and complex designs, identification of the appropriate level for tests and full reporting of outcomes |
| ☒ | ☐ | Estimates of effect sizes (e.g. Cohen's *d*, Pearson's *r*), indicating how they were calculated |

*Our web collection on statistics for biologists contains articles on many of the points above.*

## Software and code

Policy information about availability of computer code

| | |
|---|---|
| Data collection | Peptides were analyzed on an Orbitrap Fusion Tribrid mass spectrometer (Thermo Fisher). Ribosomal footprints were sequenced on an Illumina NextSeq 500. Cells were counted by EVE Automated Cell Counter (NanoEnTek). OXPHOS data were collected on an Indiko automated photometer (Thermo Fisher Scientific). |
| Data analysis | Proteomics data were analyzed using MaxQuant, version 1.6.17.0. Differential expression analysis was performed using limma, version 3.34.9 in R, version 3.4.3. Sequencing data were analyzed using the MitoRiboSeq analysis pipeline (PMID: 33953394) (open-source software and custom Python and R codes). Pooled sequencing data was split by bcl2fastq conversion software (Illumina). 3'-adapters were trimmed using Cutadapt (DOI: 10.14806/ej.17.1.200). Sequences were aligned using Burrows-Wheeler Aligner (BWA) (DOI: 10.1093/bioinformatics/btp698). A-sites were identified using Plastid (DOI: 10.1186/S12864-016-3278-X). Image analysis was performed in ImageJ version 1.52. Statistical analysis was performed using GraphPad Prism version 9.4.0. |

For manuscripts utilizing custom algorithms or software that are central to the research but not yet described in published literature, software must be made available to editors and reviewers. We strongly encourage code deposition in a community repository (e.g. GitHub). See the Nature Portfolio guidelines for submitting code & software for further information.

## Data

Policy information about availability of data

All manuscripts must include a data availability statement. This statement should provide the following information, where applicable:

- Accession codes, unique identifiers, or web links for publicly available datasets
- A description of any restrictions on data availability
- For clinical datasets or third party data, please ensure that the statement adheres to our policy

Ribosome profiling data are deposited at array express under accession number: E-MTAB-11687. The mass spectrometry proteomics data have been deposited to the ProteomeXchange Consortium via the PRIDE 46 partner repository with the dataset identifier PXD034342.

## Human research participants

Policy information about studies involving human research participants and Sex and Gender in Research.

| | |
|---|---|
| Reporting on sex and gender | N/A |
| Population characteristics | N/A |
| Recruitment | N/A |
| Ethics oversight | N/A |

Note that full information on the approval of the study protocol must also be provided in the manuscript.

# Field-specific reporting

Please select the one below that is the best fit for your research. If you are not sure, read the appropriate sections before making your selection.

☒ Life sciences ☐ Behavioural & social sciences ☐ Ecological, evolutionary & environmental sciences

For a reference copy of the document with all sections, see nature.com/documents/nr-reporting-summary-flat.pdf

# Life sciences study design

All studies must disclose on these points even when the disclosure is negative.

| | |
|---|---|
| Sample size | Sample sizes were determined based on field standards for obtaining sufficient statistical power (at least 3 biological replicates). Exact sample sizes are depicted in Figure legends. |
| Data exclusions | No data were excluded from the analysis. |
| Replication | Two different knockout cell lines for the same gene were analyzed . Biochemical experiments including quantitative mass spectrometry, northern blotting, de novo mitochondrial translation assay, protein steady state levels, OXPHOS activity assay and in vitro translation assay were performed at least three times. All attempts in replication were successful. |
| Randomization | Positive clones from MTRF1 knockout generation were randomly chosen. Experiments were performed without randomization as this is not applicable for biochemical characterization of cell lines. |
| Blinding | Blinding was not relevant to this study. For biochemical characterization of cell lines the investigator has to be aware of the sample identity. |

# Reporting for specific materials, systems and methods

We require information from authors about some types of materials, experimental systems and methods used in many studies. Here, indicate whether each material, system or method listed is relevant to your study. If you are not sure if a list item applies to your research, read the appropriate section before selecting a response.

## Materials & experimental systems

| n/a | Involved in the study |
|---|---|
| ☐ | ☒ Antibodies |
| ☐ | ☒ Eukaryotic cell lines |
| ☒ | ☐ Palaeontology and archaeology |
| ☒ | ☐ Animals and other organisms |
| ☒ | ☐ Clinical data |
| ☒ | ☐ Dual use research of concern |

## Methods

| n/a | Involved in the study |
|---|---|
| ☒ | ☐ ChIP-seq |
| ☒ | ☐ Flow cytometry |
| ☒ | ☐ MRI-based neuroimaging |

# Antibodies

| | |
|---|---|
| Antibodies used | All antibodies used in this study are listed in detail in Supplementary Table 2. |
| Validation | Information on validation of primary antibodies against following antigenes: MRPL3 - validated by the manufacturer (in Western blot using lysates of human cell lines RT-4 and U-251 MG sp); MRPS15 - validated by the manufacturer (in Western blot using lysates of human cell lines HeLa, MCF-7, Raji, in IP experiment from mouse brain); MRPS15 - validated by the manufacutrer (in Western blot using lysates of human cell lines HeLa, MCF-7, Raji, in IP experiment from mouse brain);<br>OXPHOS cocktail human - validated? by the manufacturer (in Western blot using lysates of human B cell lymphoma lines);<br>SDHA - validated by the manufacturer (in Western blot using lysates of wild-type and SDHA-deficient SH-SY5Y cell lines);<br>COX1 - validated by the manufacturer (in Western blot using lysates of SH-SY5Y cell lines);<br>ND6 - validated by the manufacturer (in Western blot using lysates of HepG2 cell lines and mouse heart, mouse brain, mouse liver, rat brain tissues); vinculin - validated by the manufacturer (in Western blot using lysates of various cell lines and tissues);<br>beta-actin - validated by the manufacturer (in Western blot using lysates of HeLa, Jurkat, A431, HEK293, NIH 3T3, PC12 cell lines);<br>NDUFB10 - validated by the manufacturer (in Western blot using lysates of HeLa, HepG2, Jurkat cell lines as well as in IP experiment from HeLa cell extract);<br>NDUFA9 - validated by the manufacturer (in Western blot using lysates of various cell lines and tissues (total and mitochondrial extracts));<br>COX2 - validated by the manufacturer (in Western blot using lysates of mitochondria from human heart tissue);<br>COX5a - validated in the study analyzing OXPHOS complexes of known sizes by native PAGE (Fig. 3e);<br>mtRF1 - validated in the study (in Western blot, by comparing wild-type HEK293 line lysate with those of generated mtRF1-deficient and -overexpressing lines (Fig. 1b, 4d); |

# Eukaryotic cell lines

Policy information about cell lines and Sex and Gender in Research

| | |
|---|---|
| Cell line source(s) | Flp-In TREx cell line was purchased from Thermofisher Scientific (catalog number: R78007). |
| Authentication | Generation of MTRF1 knockout cell lines was validated by sequencing. |
| Mycoplasma contamination | Cell line tested negative for mycoplasma contamination. |
| Commonly misidentified lines<br>(See ICLAC register) | No commonly misidentified cell lines were used in the study. |
